# Supplementary material for: Patterns of Health and Health Service Use in a Prospective Cohort of Aboriginal and Torres Strait Islander Children Aged 5–9 Years Living in Urban, Regional and Remote Areas of South Australia
Source: Int J Environ Res Public Health. 2023 Jun 19;20(12):6172. doi: 10.3390/ijerph20126172 (PMC10298277; doi:10.3390/ijerph20126172)
Supplement: Supplementary file 1 [file ijerph-20-06172-s001.zip › ijerph-2311666-supplementary.pdf]

Supplementary Table S1. Characteristics of families participating in Waves 1 and 2 of the Aboriginal Families Study.

	Wave 1 (n= 344) n (%)	Wave 2 (n= 246) n (%)
**Mothers' age at birth of study child**		
15-19 years	55 (16.0)	36 (14.6)
20-24 years	140 (40.7)	99 (40.2)
25-29 years	91 (26.5)	67 (27.2)
30 - 35 years	33 (9.6)	27 (11.0)
> 35 years	25 (7.3)	17 (6.9)
**Mothers' indigenous status**		
Aboriginal and/or Torres Strait Islander	319 (92.7)	226 (91.9)
Not Aboriginal or Torres Strait Islander[1]	25 (7.3)	20 (8.1)
**Birth weight of study child**		
<2,500 grams	49 (15.0)	31 (13.4)
≥2,500 grams	278 (85.0)	201 (86.6)
**Gestation at birth of study child**		
≥37 weeks	278 (86.9)	198 (86.1)
<37 weeks	42 (13.1)	32 (13.9)
**Study child born small for gestational age**		
≥10th percentile	240 (79.7)	174 (80.9)
<10th percentile	61 (20.3)	41 (19.1)
**Mothers' place of residence at wave 1**		
Metropolitan area	134 (39.0)	99 (40.2)
Regional	123 (35.8)	90 (36.6)
Remote	87 (25.3)	57 (23.2)
**Mothers' highest educational qualification at wave 1**		
University degree	22 (6.4)	19 (7.7)
Diploma/certificate	155 (45.1)	119 (48.4)
Year 12	33 (9.6)	23 (9.3)
Less than Year 12	134 (39.0)	85 (34.6)
**Mothers' participation in paid employment at wave 1**		
Full time job	60 (17.4)	32 (13.0)
Part time job	39 (11.3)	50 (20.3)
Not in paid employment	245 (71.2)	164 (66.7)
**Stressful events/social health issues during pregnancy**		
None	45 (13.1)	30 (12.2)
One to two	105 (30.6)	77 (31.3)
Three or more	193 (56.3)	139 (56.5)
**Total**	344 (100.0)	246 (100.0)

[1] Non-Aboriginal mothers are mothers of Aboriginal children
